# Supplementary material for: Molecular Mechanisms Underlying the Elevated Expression of a Potentially Type 2 Diabetes Mellitus Associated SCD1 Variant
Source: Int J Mol Sci. 2022 Jun 2;23(11):6221. doi: 10.3390/ijms23116221 (PMC9181825; doi:10.3390/ijms23116221)
Supplement: Supplementary file 1 [file ijms-23-06221-s001.zip › ijms-1733470-supplementary.pdf]

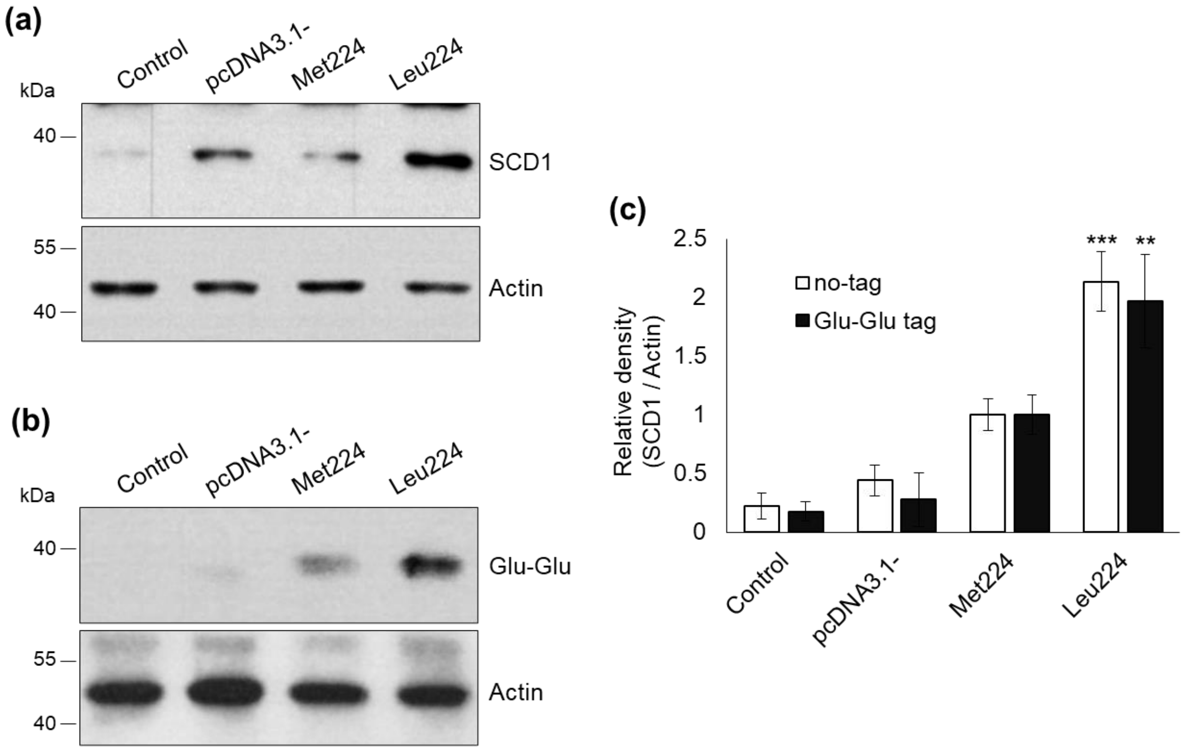

Figure S1. Expression of M224L variants of stearoyl-CoA desaturase-1 in transiently transfected HepG2 cells. HepG2 cells were harvested and processed 24 h after transfection. Aliquots of cell lysates (5 µg) were loaded on 12% SDS-polyacrylamide gel, transferred to Immobilon-P membrane and SCD1 was detected with an anti-SCD1 (**a**) and an anti-Glu-Glu tag (**b**) antibodies, respectively. Actin was measured as loading control. Representative immunoblots of three independent experiments are shown. The band intensities were quantitated by densitometry and SCD1/Actin ratios are shown as bar graphs (**c**). Data are shown as mean values ± S.D. Statistical analysis was performed with the Tukey-Kramer Multiple Comparisons Test. $^{**}p < 0.01$; $^{***}p < 0.001$.

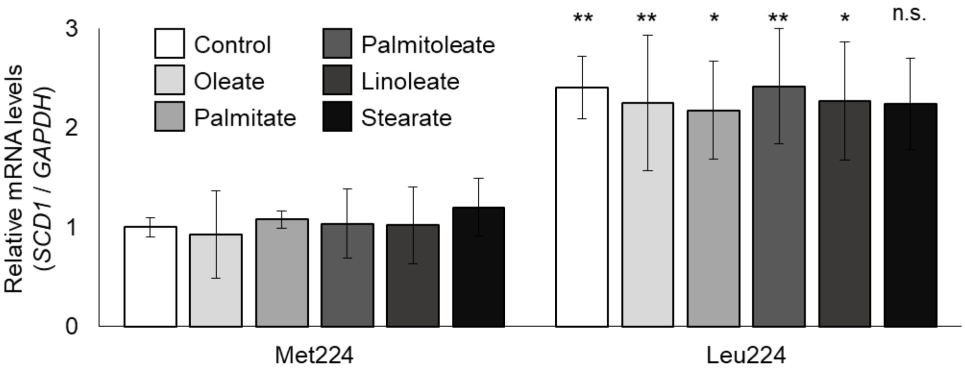

Figure S2. Effect of various fatty acids on the mRNA expression of M224L SCD1 variants in HEK293T cells. qPCR was carried out using GAPDH and SCD1-Glu-Glu tag sequence specific primers as indicated in *Materials and Methods*. The diagram presented depicts the results of three independent measurements. Statistical analysis was performed with the Tukey-Kramer Multiple Comparisons Test. Data are shown as mean values ± S.D. $^{*}p < 0.05$; $^{**}p < 0.01$.; n.s.: non-significant difference

Table S1. Intracellular amount of C16:0, C16:1, C:18:0 and C18:1 fatty acids in M224L SCD1 overexpressing HEK293T cells.

	Amount of fatty acid (μg/mg)											
	C16:0 (palmitate)			C16:1 (palmitoleate)			C18:0 (stearate)			C18:1 (oleate)		
**Control**	25.20	±	1.29	11.52	±	0.42	11.48	±	0.52	32.27	±	1.36
**pcDNA3.1-**	25.39	±	0.91	11.02	±	0.37	12.21	±	0.74	31.66	±	1.62
**Met224**	27.11	±	3.74	18.73	±	2.23	12.50	±	1.87	37.95	±	5.20
**Leu224**	40.36	±	7.74	29.30	±	5.77	18.77	±	3.48	70.83	±	12.86
